# Dextran-Coated Iron Oxide Nanoparticles Loaded with 5-Fluorouracil for Drug-Delivery Applications

**DOI:** 10.3390/nano13121811

**Published:** 2023-06-06

**Authors:** Daniela Predoi, Mihaela Balas, Madalina Andreea Badea, Steluta Carmen Ciobanu, Nicolas Buton, Anca Dinischiotu

**Affiliations:** 1 National Institute of Materials Physics, Atomistilor Street, No. 405A, P.O. Box MG 07, 077125 Magurele, Romania; ciobanucs@gmail.com; 2 Department of Biochemistry and Molecular Biology, Faculty of Biology, University of Bucharest, 91-95 Splaiul Independentei, 050095 Bucharest, Romania; madalina-andreea.badea@bio.unibuc.ro (M.A.B.); anca.dinischiotu@bio.unibuc.ro (A.D.); 3 Research Institute of the University of Bucharest (ICUB), University of Bucharest, 90-92 Sos. Panduri, 050663 Bucharest, Romania; 4 HORIBA Jobin Yvon S.A.S., 6-18, Rue du Canal, CEDEX, 91165 Longjumeau, France; nicolas.buton@horiba.com

**Keywords:** iron oxide nanoparticles, dextran, 5-Fluorouracil, Caco-2 cells, toxicity, drug-delivery system, anti-proliferative effect

## Abstract

This study aims to design and test different formulations composed of dextran-coated iron oxide nanoparticles (IONPs) loaded with 5-Fluorouracil (5-FU) with varying nanoparticle:drug ratios on colorectal cancer cells. The stable suspension of IONPs s was synthesized by the adapted co-precipitation method. The stable suspension of IONPs was mixed with a solution of dextran and 5-FU solubilized in a saline solution. The final suspensions with optimized ratios of IONP:5-FU in the final suspension were 0.5:1, 1:1, and 1.5:1. The information on the morphology and size distribution of the IONPs suspension and IONP loads with 5-FU was obtained using scanning electron microscopy (SEM). The presence of 5-FU and dextran on the surface of the IONPs was highlighted by energy-dispersive X-ray spectroscopy (EDS) studies. The determination of the surface charge of the nanoparticles in the final suspensions of IONP:5-FU was achieved by measuring the zeta potential (ζ). The hydrodynamic diameter of the resulting suspensions of IONP:5-FU was determined by dynamic light scattering (DLS). A cytocompatibility analysis was performed using Caco-2 (human epithelial colorectal adenocarcinoma) cells. In this research, our goal was to find a relationship between the formulation ratio of nanoparticles and drug, and the cellular response after exposure, as a strategy to increase the efficacy of this drug-delivery system. The nanoparticle uptake and antitumor activity, including modulation of oxidative stress, apoptosis, and proliferation biomarkers, were analyzed. The present study showed that the nanoformulation with the ratio IONP:5-FU 1.5:1 had the highest anti-tumor efficiency. Moreover, decreased MCM-2 expression in Caco-2 cells exposed to dextran-coated iron oxide nanoparticles loaded with 5-FU was demonstrated for the first time.

## 1. Introduction

Colorectal cancer (CRC) is the third most prevalent malignant tumor and the second most prevalent cause of cancer death in the world. Generally, CRC occurs in people older than 50 [1], more in men than in women [2], and in those who have a family history of CRC [3] as well as inflammatory bowel disease [4]. Overweight or obesity [5], low physical exercise [6], excess consumption of red meat and processed meat, diets low in fiber, calcium, milk, and whole grains [7], smoking [8], and alcohol use [9] are risk factors for this pathology. Globally, it is predicted that the total number of new CRC cases will be about 3.2 million in 2040, due to population aging, exposure to environmental risk factors, and unhealthy lifestyles [10].

Surgical resection is the standard treatment for patients with CRC, followed by adjuvant chemotherapy [11]. Current chemotherapy frequently involves 5-Fluorouracil (5-FU), an inhibitor of thymidylate synthase, which impairs DNA replication, exerting an antitumor effect. For increasing efficacy, 5-FU can be combined with oxaliplatin or irinotecan [12]. This antimetabolite is hydrosoluble, enters the cells through the uracil transporter, and is transformed in a reaction catalyzed by dihydropyrimidine dehydrogenase (DPD) to the inactive metabolite dihydrofluorouracil. DPD is highly expressed in liver and cancer cells and is involved in 5-FU resistance, which generates the necessity to increase the applied doses [13]. However, 5-FU results in cardiotoxicity in a net duration of therapy-dependent manner [14], manifested by anginal chest pains, palpitations, and dyspnea [15], and generates adverse reactions at the level of the gastrointestinal system, blood components, and skin [16].

In this context, new formulations of 5-FU to diminish the side effects and increase the therapeutic efficacy are needed. Several drug-delivery systems have been achieved, including those based on inorganic or organic nanoparticles [17]. Several types of organic nanoparticles based on chitosan-graft-poly (ε caprolactone) [18], chitosan and polyaspartic acid [19], or bovine serum albumin [20] have been developed. In contrast to organic nanocarriers, the inorganic ones present good stability in the biological environment and considerable loading efficiency. Multiple-walled nanotubes have been used for different antineoplastic drug-delivery systems [21,22] with good efficiency against cancer cells. Also, different formulations of 5-FU with iron oxide nanoparticles (IONPs), with high toxicity in a magnetic field [23], and polymer-coated magnetite nanographene oxide nanoparticles that can be used as a theragnostic agent and in magnetic hyperthermia [24] have been achieved. Still, the poor loading capacity of nanoparticles and their dose-limiting toxicity remain the major limitations of drug-delivery nanosystems. Previous studies conducted by other researchers pointed out that formulation design may significantly affect the interaction of nanosystems with molecules, cellular uptake, drug delivery, drug encapsulation, or the efficacy of the target application [25,26]. Thus, controlling the factor ratio between the components in formulations of drug-delivery nanosystems might enhance their properties to maximize the anti-tumoral effects on colon cancer cells. The study of the effects of the optimized formulations on cancer cells can be highly valuable and worth exploring to understand the factors involved in their functionality.

The aim of our study was to design and test different formulations composed of dextran-coated IONPs loaded with 5-FU with varying nanoparticle: drug ratios on colorectal cancer cells. Our focus was to find a relationship between the formulation ratio of nanoparticles and drug and the cellular response resulting after exposure as a strategy to increase the efficacy of the drug-delivery system. This study demonstrates also, for the first time, the decrease in MCM-2 expression in Caco-2 cells exposed to dextran-coated IONPs loaded with 5-FU.

## 2. Materials and Methods

### 2.1. Synthesis and Characterization of the Drug-Delivery System

#### 2.1.1. Materials

For the development of dextran-coated IONPs, the next precursors were used: ferrous chloride tetrahydrate (FeCl_2_⋅4H_2_O), ferric chloride hexahydrate (FeCl_3_⋅6H_2_O), sodium nitrate (NaNO_3_), perchloric acid (HClO_4_), hydrochloric acid (HCl), and sodium hydroxide (NaOH). All the precursors were purchased from Merck (Rahway, NJ, USA). Both double-distilled water and deionized water were used for the sample-rinsing and in the synthesis process.

#### 2.1.2. Dextran-Coated IONPs Loaded with 5-FU

Dextran-coated IONPs loaded with 5-FU were obtained by adapting Massart’s co-precipitation method [27] in accordance with our previous studies [28,29,30,31,32]. In summary, a mixture of ferrous chloride tetrahydrate, ferric chloride hexahydrate, and a solution of NaNO_3_ (1 mol/L^−1^) was added dropwise under vigorous stirring to a NaOH solution (2 mol L^−1^) [28,29,30,31,32]. Finally, the suspensions were separated by centrifugation and washed in double-distilled water [28,29,30,31,32]. The IONPs were then dispersed in deionized water.

The stable suspension of IONPs was mixed with a solution of dextran (10%) and 5-FU solubilized in a saline solution. The mixture was made at room temperature under continuous stirring for 24 h, and then separated and washed with deionized water in order to eliminate any unloaded drug. Three different ratios of IONPs and 5-FU, including sample 3 (IONP:5-FU 0.5:1), sample 4 (IONP:5-FU 1:1), and sample 5 (IONP:5-FU 1.5:1), were analyzed. The suspensions of IONPs, named sample 1, and 5-FU, named sample 2, were also used in this study.

#### 2.1.3. Physiochemical Characterization

Information regarding the sample’s morphology and chemical composition was obtained with the aid of scanning electron microscopy (SEM) and energy-dispersive spectroscopy (EDX). The studies were carried out using a Hitachi S4500 scanning electron microscope (Hitachi, Tokyo, Japan) equipped with an energy-dispersive X-ray (EDX) detection system (Ametek EDAX Inc., Mahwah, NJ, USA) operating at 20 kV. In order to analyze the morphology and chemical composition of the samples by SEM and EDS techniques, one drop of each suspension was put on the microscope sample holder and dried. Afterward, the sample holder was inserted into the microscope and the sample was analyzed.

The data regarding the particle size analysis, as well as information about the zeta potential of the samples, were investigated with an SZ-100 Nanoparticle Analyzer instrument from Horiba Jobin Yvon (Horiba, Ltd., Kyoto, Japan). The particle size analysis was performed by dynamic light scattering (DLS). Both DLS and zeta potential (ζ) measurements were recorded at room temperature. Three determinations were recorded for each sample. The zeta potential of the samples was measured using diluted samples in water.

#### 2.1.4. Cell Culture and Treatment

Human colorectal adenocarcinoma cells (Caco-2 cell line, HTB-37) from ATCC/LGC Standards GmbH (Wesel, Germany) were grown in MEM medium (61100-87, Gibco by Life Technologies, Carlsbad, CA, USA) supplemented with 1.5 g/L of NaHCO_3_, 1 mM of sodium pyruvate, 20% fetal bovine serum (10270-106, origin South America, Gibco, by Life Technologies, Carlsbad, CA, USA), and 1% antibiotic-antimycotic solution (A5955; Sigma-Aldrich, St. Louis, MO, USA). The cell culture was maintained in a humidified atmosphere at 37 °C and 5% CO_2_. For treatment, the Caco-2 cells were seeded at a 5 × 10^4^ cells/mL density and allowed to adhere overnight. The nanoformulations (IONP:5-FU = 0.5:1, 1:1, and 1.5:1) and free components (IONPs and 5-FU) were added to the culture medium in various concentrations. Doses between 0.1–100 μg/mL IONPs and 5–250 μg/mL 5-FU were applied to the Caco-2 cells for 24 and 48 h for an MTT assay. For the other tests, a dose of 200 μg/mL of 5-FU was selected, and the corresponding doses of IONPs in the nanoformulation were 6 (0.5), 12 (1), and 18 μg/mL (1.5). Untreated Caco-2 cells were used as a negative control. Before treatment, the suspensions were sterilized under UV radiation for 1 h.

#### 2.1.5. MTT Assay

Cell viability was estimated by a 3-(4,5-dimethylthiazol-2-yl)-2,5-diphenyltetrazolium bromide (MTT) spectrophotometric test. For this assay, the Caco-2 cells were seeded in a 96-well plate at a density of 10^4^ cells/well, and after 24 h they were treated with our suspension. After 24 and 48 h exposure, the medium was removed from all the wells, and a volume of 80 μL of 1 mg/mL MTT solution (M2128, Sigma-Aldrich, St. Louis, MO, USA) was added for 2 h incubation at 37 °C. The formazan produced by the MTT reduction in the metabolic active cells was solubilized in 150 μL of isopropanol, and the absorbance was measured at 595 nm using a FlexStation 3 microplate reader (Molecular Devices, San Jose, CA, USA). No interference of NPs with the MTT assay was detected for the used experimental conditions.

#### 2.1.6. Microscopic Imaging of Cell Morphology

The morpho-structural characteristics of the cells after treatment were captured by phase-contrast microscopy at a magnification of 10×. The acquisition of images was carried out with an Olympus IX73 microscope (Olympus, Tokyo, Japan) equipped with a Hamamatsu ORCA-03G camera (A3472-06, Hamamatsu, Japan) and the CellSens Dimension software (v 1.11, Olympus).

#### 2.1.7. Detection of Lactate Dehydrogenase (LDH) Leakage

The amount of LDH released in the culture medium is an indicator of the cell membrane integrity and cytotoxicity of compounds. This investigation was performed using the Cytotoxicity Detection Kit (LDH), ver. 11, (11644793001, Roche, Basel, Switzerland). Thus, a volume of 50 μL of culture supernatant, removed from the wells of the same 96-well plates used for the MTT assay, was homogenized with a volume of 50 μL of reaction mix consisting of a catalyst and dye solution (1:45) and incubated for 15 min in the dark. The absorbance was read at 490 nm using a FlexStation 3 microplate reader (Molecular Devices, San Jose, CA, USA).

#### 2.1.8. Quantification of Total Intracellular Iron

The iron content in the Caco-2 cells was measured by an Iron Assay Kit (MAK025, Sigma-Aldrich, St. Louis, MO, USA) according to the manufacturer’s instructions. Total iron (Fe^2+^ and Fe^3+^) from cell homogenate reacts with a chromogen, resulting in a colorimetric product absorbing at 593 nm, proportional to the iron present. In a 96-well plate, a volume of 100 μL of diluted sample was mixed with 5 μL of iron reducer to reduce Fe^3+^ to Fe^2+^. The microplate was incubated for 30 min at 25 °C in the dark. After the addition of 100 μL of an iron probe in each well, the microplate was incubated for another 60 min at 25 °C, protected from light. The absorbance was read at 593 nm using a FlexStation 3 microplate reader (Molecular Devices, San Jose, CA, USA). The iron was estimated using a standard curve prepared from a 1 mM standard solution.

#### 2.1.9. Measurement of H_2_O_2_ Production

The production of hydrogen peroxide (H_2_O_2_) was quantified with the Amplex Red Assay Kit (A22188, Invitrogen, Carlsbad, CA, USA) following the manufacturer’s instructions. This assay utilizes Amplex Red reagent (10-acetyl-3,7-dihydroxyphenoxazine) in combination with horseradish peroxidase (HRP) to detect H_2_O_2_ released from biological samples, including cells. Briefly, Caco-2 cells were seeded in a 96-well plate and allowed to adhere overnight. Immediately after treatment, an equal volume (50 μL) of the reaction mixture containing 100 μM of Amplex Red and 0.2 U/mL of HRP was added to each well, and the plate was incubated at 37 °C for 30 min, protected from light. The formation of the red-fluorescent oxidation product resorufin was registered at 560 nm after 24 and 48 h using a microplate reader. Wells similarly treated but without cells were used as the blank. The blank values were subtracted from the corresponding sample values to obtain the corrected absorbance.

#### 2.1.10. Immunoblotting

After treatment, the protein extract from the Caco-2 cells was obtained as previously described [33]. Equal amounts of protein (50 µg) from treated and untreated Caco-2 cells were separated through SDS–polyacrylamide gels (8% and 15% resolving gel; 4.5% concentration gel) in Tris–glycine buffer at 90 V for 2 h. The proteins were transferred from the SDS–polyacrylamide gel to a 0.45 µm pore PVDF membrane (IPVH00010, Merck Millipore, Darmstadt, Germany) using a wet transfer system. The membranes were developed using a WesternBreeze Chromogenic Kit, anti-mouse (WB7103, Invitrogen, Carlsbad, CA, USA) and primary antibodies for MCM-2 (sc-373702, Santa Cruz Biotechnology, Heidelberg, Germany), caspase-3 (sc-7272, Santa Cruz Biotechnology, Heidelberg, Germany), and β-actin (A1978, Sigma-Aldrich, St. Louis, MO, USA) proteins. The protein bands were revealed by chromogenic detection using a BCIP/NBT substrate and visualized with the ChemiDoc Imaging System (Bio-Rad, Hercules, CA, USA). Quantification of the bands on blot membranes was performed using the Image Lab software (ver. 5.2, Bio-Rad, Hercules, CA, USA). The MCM-2 and caspase-3 protein levels were normalized to β-actin (the reference protein) level and represented as percentages of control.

#### 2.1.11. Drug-Loading and Drug-Release Studies

The drug-loading studies were conducted following the procedure described by Amini-Fazl, M.S. et al. [34]. Thus, a proper IONPs quantity was added into a 10 mL solution of 5-FU (50 mg/mL) and shaken. Then, the 5-FU-loaded IONPs were washed and dried at room temperature. The collected supernatant was analyzed by UV–Vis spectrophotometry (Able Jasco, PW de Meern, The Netherlands,). The drug-loading efficiency (D_L_) was obtained with the aid of Equation (1):

(1) $$D_{L}=\;\frac{\left(\mathrm{Total}\;5\mathrm{FU}-\mathrm{Free}\;5\mathrm{FU}\right)}{\mathrm{Total}\;\mathrm{mass}\;\mathrm{of}\;\mathrm{IONPs}}\times 100$$

Further, the 5-FU drug-release experiments were made at pH 7.4 and 37 °C using a PBS buffer solution and following the procedure previously described by S. Ayyanaar et al. [35]. The drug-release efficiency was determined with the aid of a UV–Vis spectrophotometer (Able Jasco, PW de Meern, The Netherlands,).

#### 2.1.12. Statistical Analysis

All the investigations were performed in triplicate. The results were expressed as relative values in comparison with the control (100%) and calculated as the mean ± standard deviation. The data were statistically analyzed in GraphPad Prism (Version 8, GraphPad Software, La Jolla, CA, USA), using the two-way ANOVA method and Dunnett’s multiple comparisons tests (treated cells vs. control). The values *p* < 0.05 (*), *p* < 0.01 (**), and *p* < 0.001 (***) were considered significant.

## 3. Results

### 3.1. Characterization of Dextran-Coated IONP Loaded with 5-FU

**Figure 1:**
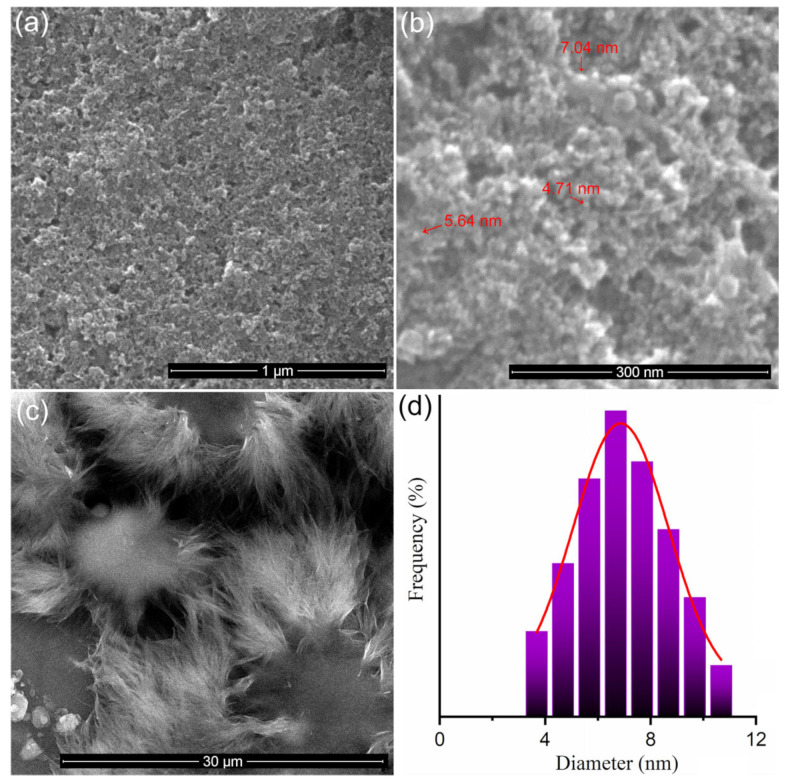
SEM images of the iron oxide nanoparticles at 50,000× magnification (**a**), at 400,000× magnification, (**b**) and 5-FU at 50,000× magnification (**c**). Size distributions of the IONPs (**d**).

Figure 1a,b reveals the SEM micrographs of IONPs at different magnifications. The particles have a nanometric size and spherical shape.

From the SEM micrograph shown in Figure 1b, the size distribution was obtained (Figure 1d) following the measurement of approximately 250 particles. The average particle size was 6.88 ± 2 nm. The normal Gaussian distribution function was used to calculate the average particle size from the particle size distribution. Figure 1c shows the SEM micrograph of sample 2 (5-FU). Thus, the 5-FU presented a morphology in the form of flakes that are arranged circularly.

**Figure 2:**
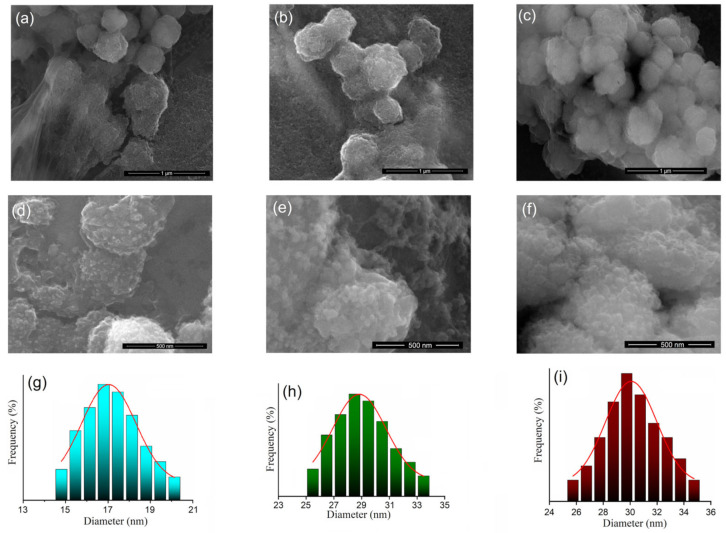
SEM image of IONP:5-FU 0.5:1 (**a**), IONP:5-FU 1:1 (**b**), and IONP:5-FU 1.5:1 (**c**) at 50,000× magnification. SEM micrographs of IONP:5-FU 0.5:1 (**d**), IONP:5-FU 1:1 (**e**), and IONP:5-FU 1.5:1 (**f**) at 500,000× magnification. Size distributions of the IONP:5-FU 0.5:1 (**g**), IONP:5-FU 1:1 (**h**), and IONP:5-FU 1.5:1 (**i**).

In Figure 2, it can be seen that the ratio between IONPs and 5-FU has a slight influence on the morphology of the particles. Figure 2a shows the SEM image of IONP:5-FU 0.5:1. Spherical particles with a nanometric size were observed. The traces of 5-FU that were not completely involved in the particle-coating process were observed (Figure 2a lower left). Figure 2b shows the SEM image of IONP:5-FU 1:1. In this case, particles with a nanometric size and spherical shape that tend to form larger spheres were observed. Figure 2c shows the SEM image of IONP:5-FU 1.5:1.

It can be seen that the size of the particles is nanometric, and the spheres formed by the nanometric particles are well-defined. High-resolution SEM micrographs of IONP:5-FU 0.5:1, IONP:5-FU 1:1, and IONP:5-FU 1.5:1 are presented in Figure 2d–f. The size distributions for the three samples are also presented in Figure 2g–i.

The size distribution of sample IONP:5-FU 0.5:1 was obtained after counting around 150 particles. The average particle size was 17.23 ± 2 nm. The size distributions of the IONP:5-FU 1:1 and IONP:5-FU 1.5:1 were obtained after counting 250 particles. The average particle size was 28.98 ± 2 nm for IONP:5-FU 1:1 and 30.19 ± 2 nm for IONP:5-FU 1.5:1.

**Figure 3:**
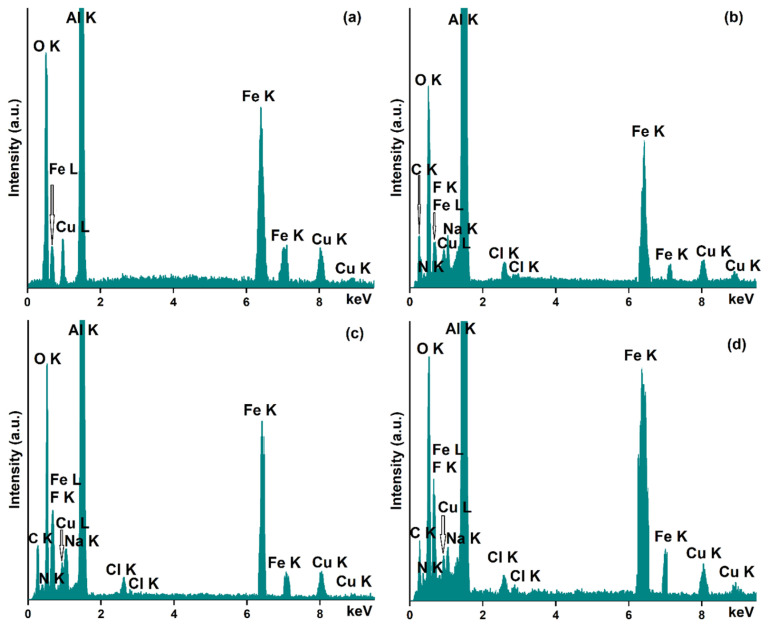
EDS spectra obtained for IONPs (**a**), IONP:5-FU 0.5:1 (**b**), IONP:5-FU 1:1 (**c**), and IONP:5-FU 1.5:1 (**d**).

The results of the EDS studies conducted on the free IONPs and the IONP loaded with 5-FU are depicted in Figure 3a–d.

As can be seen, in all the obtained EDS spectra, the presence of the constituent chemical elements of the sample holder (Al (aluminum) and Cu (copper)) on which the suspension was analyzed is highlighted. The purity of the iron oxide sample is proved by the absence of additional maxima in its EDS spectra (Figure 3a). Therefore, in the recorded EDS spectrum, only the presence of O, Fe, and C was observed, these being the main chemical elements that are found in the composition of the analyzed sample.

In the case of samples that are loaded with 5-FU (IONP:5-FU 0.5:1, IONP:5-FU 1:1, and IONP:5-FU 1.5:1), the recorded EDS spectra prove its presence in the samples. The chemical elements that belong to the 5-FU composition are C (carbon), N (nitrogen), F (fluorine), and O (oxygen). All these chemical elements can be easily observed in the EDS spectra of the samples presented in Figure 3b–d. Additionally, in Figure 3b–d, the presence of the C (carbon), Fe (iron), and O (oxygen) is attributed to the composition of the IONP. Moreover, in the recorded EDS spectra for the IONP:5-FU 0.5:1, IONP:5-FU 1:1, and IONP:5-FU 1.5:1 are also observed peaks corresponding to Na and Cl, these being mainly attributed to the chemical composition of the saline solution (used in the process of obtaining samples).

DLS and zeta potential (ζ) studies were performed to evaluate the surface charge and colloidal stability of the dextran-coated IONPs loaded with 5-FU samples. For IONP:5-FU 0.5:1, IONP:5-FU 1:1, and IONP:5-FU 1.5:1, the hydrodynamic diameters and zeta potential were analyzed as a function of time. For these studies, the samples were kept in the incubator at a constant temperature of 37 °C. The hydrodynamic diameters (D_h_) of IONP:5-FU 0.5:1, IONP:5-FU 1:1, and IONP:5-FU 1.5:1 at different storage times (1, 7, 14, and 28 days) are presented in Table 1. It is observed that the D_h_ of the same suspension after 7, 14, and 28 days increased for each analyzed sample.

The increase in D_h_ suggests that a small degree of particle agglomeration occurred after prolonged storage. However, even after 28 days, no agglomeration or sedimentation of the nanoparticles was visually observed. On the other hand, it was observed that for all three analyzed samples, the particle sizes obtained from the DLS analysis were larger than those obtained from the SEM studies. This difference can be explained by the fact that what is measured by DLS represents the hydrodynamic dimension. This hydrodynamic size represents the size of the particles surrounded by a diffuse layer, while the size obtained from the SEM studies is the size of IONPs.

For a good evaluation of the stability of the suspensions, the measurement of the ζ is very important [36]. In agreement with previous studies carried out by Laurent et al. [36], nanoparticles tend to agglomerate and are considered unstable when the ζ is between −10 mV and +10 mV. Honary et al. [37], in their studies on the effect of ζ on the properties of nano-drug-delivery systems, showed that suspensions with ζ ~ ±20 mV have short-term stability, and, when ζ < ±5 mV, the particles rapidly precipitate, forming aggregates. On the other hand, recent studies have shown that there is good stability of suspensions when ζ ≥ 30 mV and ≤60 mV in absolute value [38]. Moreover, Gumustas et al. [39] showed that, when ζ ≥ ±30 mV, we have monodisperse formulations without aggregates. The values of the ζ for the same suspension after 1, 7, 14, and 28 days of storage are presented in Table 2.

### 3.2. Evaluation of the Anti-Tumoral Activity

**Figure 4:**
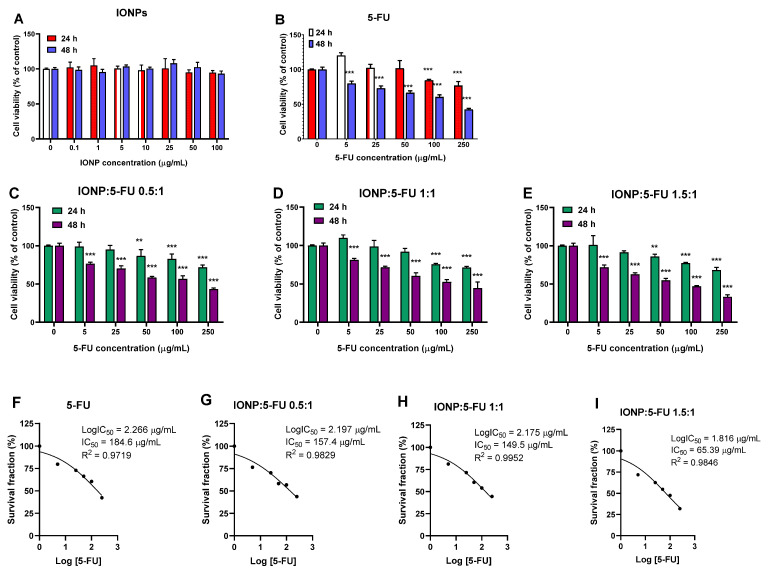
Cytotoxicity of nanoformulations in Caco-2 cells. The graphs illustrate the percentages of cell viability after 24 and 48 h exposure to (**A**) IONPs alone; (**B**) free 5-FU; (**C**) IONP:5-FU 0.5:1; (**D**) IONP:5-FU 1:1; (**E**) IONP:5-FU 1.5:1. IC_50_ values for each condition are represented at points (**F**–**I**). Untreated cells (0 μg/mL) were used as a control. Results (control vs. sample) were significant at *p* < 0.01 (**), and *p* < 0.001 (***). Error bars reflect the standard deviation.

The cytotoxic effects of IONP:5-FU nanoformulations on human colorectal cancer cells were examined 24 and 48 h after exposure by an MTT cell viability assay. First, to demonstrate the potential utility of IONPs as a biocompatible drug-delivery vehicle, free IONPs were tested on Caco-2 cells at doses of 0–100 μg/mL. We showed that the IONPs caused no decrease in cell viability at any of the tested doses (Figure 4A).

The drug 5-FU alone exhibited cytotoxic effects depending on time and dose (Figure 4B). At equivalent concentrations of 5-FU (0–250 μg/mL), the different nanoformulations reduced the cell viability of the colorectal cancer cells in a similar manner. IONP:5-FU 1.5:1 was the most cytotoxic nanoformulation, registering the highest decrease of cell viability, by 68% after 48 of exposure to a dose corresponding to 250 μg/mL of 5-FU. Compared to free 5-FU, all the nanoformulations presented enhanced cytotoxicity and reduced half-maximal inhibitory concentrations (IC50) overall. The IC50 values for 5-FU were estimated by nonlinear regression curve fitting of the MTT data for cells exposed to treatment for 48 h (Figure 4F–I). According to these results, the IC50 values registered for our nanoformulations were 157.4 μg/mL for IONP:5-FU 0.5:1, 149.5 μg/mL for IONP:5-FU 1:1, and 65.39 μg/mL for IONP:5-FU 1.5:1, which were lower than the IC50 value calculated for the free drug (184.6 μg/mL).

The IONPs probably enhanced the delivery of 5-FU to the Caco-2 cells, and, upon internalization, the drug was released inside the cells. This might explain the enhanced cytotoxicity of nanoformulations compared to 5-FU alone, due to the synergistic anti-proliferative activities on colorectal cancer cells. Our findings are similar to those of Ebadi et al. (2019) [40] that proved that magnetite nanoparticles coated with polyethylene glycol and 5-FU decreased the viability of HepG2 cells compared to the free drug. The IONPs can enter cells mainly through endocytosis mechanisms [41]. Once inside the lysosomes, the low pH environment, around pH 4–5, could dissolve dextran-coated IONPs, as previously proved [42], releasing 5-FU into the cytoplasm of Caco-2 cells. It is also possible that weakly bound drugs enter the cancer cell in their free form after their release from the surface of NPs outside the cells. The observed time-dependent cytotoxicity of the nanoformulations may suggest a gradual release of 5-FU from NPs.

**Figure 5:**
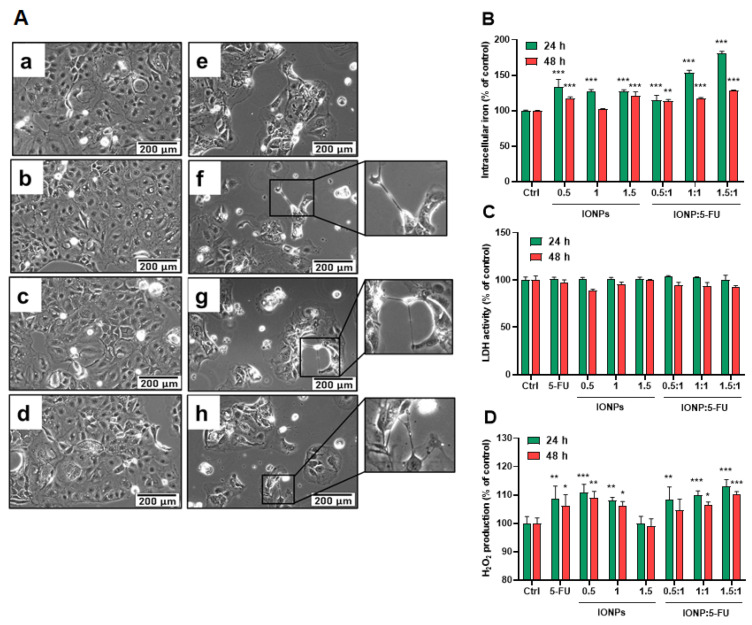
Cellular internalization of IONPs and anti-tumoral effects on Caco-2 cells. Cells were exposed for 24 and 48 h to the tested suspensions containing 5-FU in a concentration of 200 μg/mL and IONP 0.5, 1, and 1.5 with the corresponding concentrations of 6, 12, and 18 μg/mL, respectively. The figure shows (**A**) alterations of cell morphology after exposure of Caco-2 cells after 48 h to (**a**). 0 μg/mL (control), (**b**). 6 μg/mL of IONP, (**c**). 12 μg/mL of IONP, (**d**). 18 μg/mL of IONP, (**e**). 200 μg/mL of 5-FU, (**f**). 6:200 μg/mL of IONP:5-FU, (**g**). 12:200 μg/mL of IONP:5-FU, (**h**). 18:200 μg/mL of IONP:5-FU. The zoomed images present suggestive cell morphological modifications, (**B**) intracellular iron content, (**C**) LDH leakage, and (**D**) H_2_O_2_ production. Results (control vs. sample) were significant at *p* < 0.05 (*), *p* < 0.01 (**), and *p* < 0.001 (***). Error bars reflect the standard deviation.

To further investigate the internalization of IONPs, the Caco-2 cells were treated with the corresponding IONP doses for 200 μg/mL of 5-FU (approximation of the IC50 value for 5-FU) in the tested suspensions. The evaluation of total intracellular iron content demonstrated the uptake of IONPs into the cytosol after 24 h of exposure and a decline in their level after 48 h (Figure 5B). Free IONPs at similar doses to the nanoformulations registered less internalization, probably due to the formation of particle aggregates in the absence of 5-FU, which prevented the NP cellular internalization. Caco-2 cells uptake IONPs in a higher percentage when these are associated with 5-FU. At a ratio of IONP:5-FU 1:1 and 1.5:1, the cell uptake increases by 26% and 53%, respectively, compared to free IONPs at the same concentration. However, when 5-FU was found in a higher proportion than IONPs in the suspension (ratio 0.5:1), the cell uptake decreased by 18%. Probably, the intracellular iron concentration decreased after 48 h due to the exocytosis of IONPs. As previously reported, IONPs may be subjected to exocytosis starting within 24 h and continuing after 72 h [43]. IONPs trapped in endosomes may be transported to the cell periphery and then expelled from cells through exocytosis [44].

Furthermore, in order to see how our nanoformulations exerted their toxic effects, the morphology of Caco-2 cells, membrane integrity, and oxidative potential were evaluated. Microscopic examination of Caco-2 cells after exposure revealed a reduced density of cells and the appearance of atypic morphologic features consisting of cell shrinkage, extension/retraction of membranes, and cell detachment (Figure 5A). However, no LDH release was detected in any of the conditions, indicating that the membrane integrity was not altered.

Enhanced production of H_2_O_2_ was found in Caco-2 cells treated with free 5-FU and IONPs as well as those treated with the nanoformulations. As seen in Figure 5D, the levels of H_2_O_2_ in the cells treated with the nanoformulations did not exceed the ones found in the cells exposed to individual components. The estimation of H_2_O_2_ production is probably not sufficient to evaluate the overall induction of reactive oxygen species (ROS) by IONPs, due to the decomposition of H_2_O_2_ in the Fenton reaction in the presence of ferrous ions (Fe^2+^). As is known, after internalization, IONPs reach the lysosomes, where they are digested into Fe^2+^ and Fe^3+^ ions that react with H_2_O_2_, leading to the generation of highly reactive hydroxyl radical (HO•) and hydroperoxyl radical (HOO•) respectively. Other species such as superoxide ions (O_2_•^−^) and singlet oxygen (_1_O^2^) may also be generated by iron ions, thereby increasing the intracellular ROS levels [45]. Magnetite (Fe_3_O_4_) and maghemite (ɣ-Fe_2_O_3_) can show different cellular responses because of their ability to undergo oxidation/reduction reactions. In magnetite nanoparticles, iron is present as a mixture of ferrous iron (Fe^2+^) and ferric iron (Fe^3+^) ions, while, in maghemite, iron ions are mostly in the ferric state. Thus, maghemite has less oxidative power, producing fewer radicals compared to magnetite [46]. Upon internalization, part of ɣ-Fe_2_O_3_ NPs participates in the Haber–Weiss cycle. Initially, they are decomposed into Fe^3+^ ions in the acidic lysosome and then diffuse into the cytoplasm. Subsequently, ferric ions may react in the mitochondria with superoxide anions (O_2_•^−^), resulting in oxygen and ferrous iron formation. Then, ferrous iron can react with hydrogen peroxide, forming hydroxyl radicals (•OH), hydroxyl anions (OH^−^), and ferric iron via the Fenton reaction.

Endogenous sources of H_2_O_2_ include NADPH oxidases, the mitochondrial respiratory chain, and other oxidases (membrane-bound or free). In the cytoplasm, superoxide ions lead to the formation of H_2_O_2_ through the catalytic activity of Cu-Zn superoxide dismutase (SOD1), and, at the plasma membrane level, H_2_O_2_ is generated under the action of SOD3 and NADPH oxidases [47]. The balance of the intracellular production of ROS including H_2_O_2_ is maintained through a variety of enzymatic reactions. When overproduced, ROS may lead to the inhibition of cell growth and proliferation, oxidation of biomolecules, and eventual cell death.

**Figure 6:**
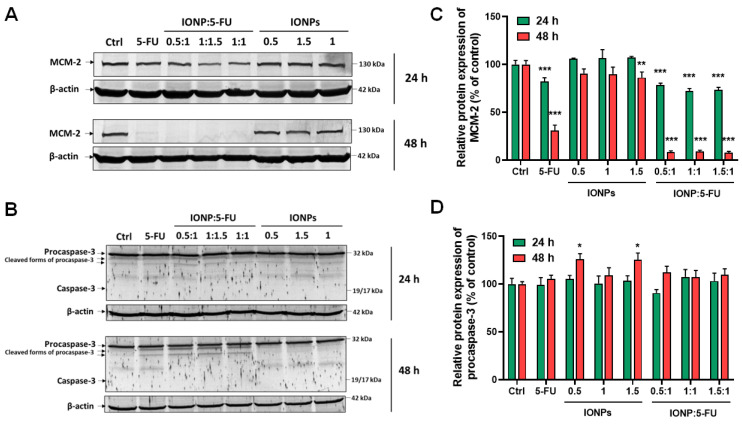
Effect of suspensions on MCM-2 and caspase-3 protein expression in Caco-2 cells. Cells were exposed for 24 and 48 h to the tested suspensions containing 5-FU in a concentration of 200 μg/mL and IONP 0.5, 1, and 1.5 with the corresponding concentrations of 6, 12, and 18 μg/mL, respectively. (**A**,**B**) present the protein bands of MCM-2, procaspase-3, caspase-3, and β-actin revealed after immunoblotting; (**C**) relative protein expression of MCM-2; (**D**) relative protein expression of procaspase-3. Results (control vs. sample) were significant at *p* < 0.05 (*), *p* < 0.01 (**), and *p* < 0.001 (***). Error bars reflect the standard deviation.

The anti-proliferative and apoptotic activity of the nanoformulations was evaluated further to shed some light on their anti-tumor potential. The protein expression of minichromosome maintenance protein 2 (MCM-2) and caspase-3 activation was analyzed by Western blot. MCM-2 is a sensitive proliferation marker, part of the MCM 2–7 complex, which acts as an essential replicative helicase for the initiation and elongation of DNA replication, playing a crucial role in cell development, proliferation, and induction of apoptosis [48]. The exposure of Caco-2 cells to the nanoformulations resulted in a statistically significant decrease in the expression of MCM-2 protein, starting within 24 h, the highest downregulation being registered for the formulation with the ratio IONP-5-FU 1.5:1. After 48 h of exposure, complete inhibition of MCM-2 protein expression was induced by all three nanoformulations (Figure 6A,C). We also demonstrated that 5-FU alone caused an important downregulation of MCM-2 in Caco-2 cells after 48 h of exposure by 69% compared to the control. The free IONPs at the same dose (18 μg/mL) as in the IONP:5-FU 1.5:1 sample decreased the MCM-2 protein expression only by 14%. Overall, the nanoformulations presented an enhanced anti-proliferative activity on human colorectal cancer cells compared to 5-FU alone.

Caspase-3 is the final executor of apoptosis resulting from the cleavage of procaspase-3. As reported in the literature, 5-FU initiates apoptosis in colorectal cancer through the activation of caspase-9 and causes the death of cells after the induction of caspase-3 [49]. In our study, Caco-2 cells did not demonstrate caspase-3 activation in all the tested conditions.

However, cleaved forms of procaspase-3 were observed on the blot membranes (Figure 6B), and a slight increase in procaspase-3 protein expression was detected after 48 h in cells treated with free IONPs (Figure 6D). The absence of caspase-3 activation might suggest a possible resistance mechanism [50], though the inhibition of proliferation caused by 5-FU could possibly facilitate autophagy [51]. Moreover, it was shown that caspase-3 can promote cancer cell growth, cellular migration, and tumor angiogenesis [40,52]; thus, its inactivity could be exploited as a therapeutic approach for colorectal cancer.

The results obtained in this study highlight the fact that 5-FU and dextran were adsorbed on the surface of IONPs. After adsorption, the surface of iron oxide nanoparticles was covered with 5-FU and dextran. IONPs coated with dextran and 5-FU have a negative charge. An increase in negative charges was observed in an iron concentration-dependent manner in the studied samples. This fact could be due to the fact that IONPs have a negative charge in a wide pH range, between 6 and 9 [53]. Furthermore, previous studies have demonstrated that by increasing the negative surface charge, an increase in drug loading is obtained while the aggregation between particles decreases [53]. Moreover, Poller et al. [54] in their studies regarding the selection of potential IONPs for breast cancer treatment based on in vitro cytotoxicity and cellular uptake studies, showed that the negative charge on the surface of magnetic particles could lead to an increase in their stability, which would reduce their aggregation in the blood. On the other hand, different parameters such as the shape or size of particles play an important role in the cellular absorption of particles. Also, cell binding processes are strongly influenced by the zeta potential associated with suspended particles [37]. Sahay et al. in their studies on the endocytosis of nanoparticles [55] showed that stronger bonds with the membrane and higher levels of cellular absorption are obtained when higher zeta potentials exist. Honary et al. [37] in their studies on the effect of zeta potential on the properties of nano-drug-delivery systems showed that the value of zeta potential is decisive because it directly influences both the stability and the release profile of the drug. Therefore, the surface charge of the particles plays a decisive role in the efficiency of nanomedicine. Our studies showed that the IONP:5-FU 1.5:1 system presented the highest tumor efficiency, which could be due to both zeta potential and iron concentration being in complete agreement with previous studies.

Our studies revealed that the IONPs’ drug-loading efficiency was about 95%. Furthermore, these results are in good agreement with the results reported by Amini-Fazl, M.S. et al. [34], which showed that the presence of iron oxide nanoparticles considerably improves the drug-loading capacity of the studied materials.

**Figure 7:**
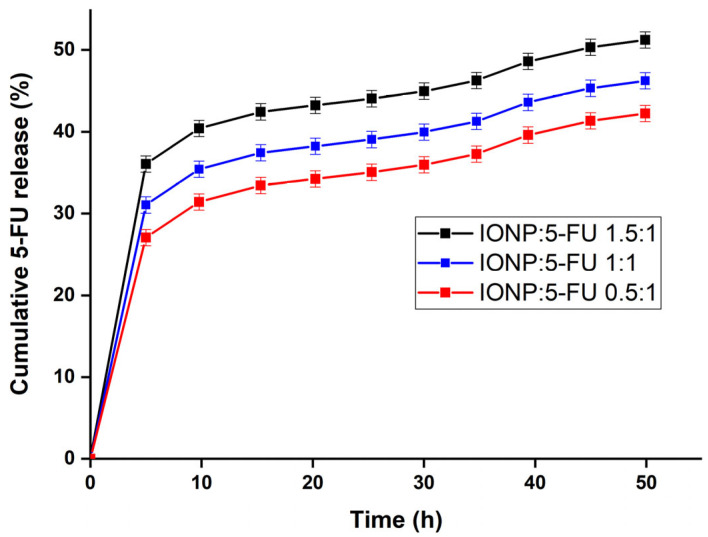
Cumulative drug-release features of the IONP:5-FU 0.5:1, IONP:5-FU 1:1, and IONP:5-FU 1.5:1 systems at pH 7.4.

Figure 7 presents the results of the drug-release features of the IONP:5-FU 0.5:1, IONP:5-FU 1:1, and IONP:5-FU 1.5:1 systems.

The results shown in Figure 7 are obtained at pH 7.4 and at a temperature of 37 °C. Our findings suggest that the best release percentage was obtained for the IONP:5-FU 1.5:1 system. Generally, it can be seen that the release percentage increases with the increase in IONPs in the studied systems. The study regarding the 5-FU drug release of CS/GO reported by Miralinaghi, P. et al. [56] showed that almost 89% of 5-FU was released in the simulated blood fluid after 5 h. Moreover, the results reported by Ayyanaar S. et al. [35] highlight that the 5-FU release percentage by the 5-FLU-MMS 1–4 was around 60% in basic release media. Also, according to the study by Amini-Fazl, M.S. et al. [34], the presence of iron oxide nanoparticles in the samples results in good drug release (5-FU) characteristics. Therefore, we could say that our results are in good agreement with the ones previously reported in the literature.

## 4. Conclusions

The aim of this study was to obtain multifunctional IONPs for use in cancer therapy to destroy cancer cells without affecting healthy cells. For this purpose, IONPs were coated with dextran and loaded with 5-FU, and then characterized and evaluated for their efficacy and suitability as delivery systems. The hydrodynamic diameter increased over time for all the analyzed systems, but the stability was not affected after prolonged storage. The values obtained for the zeta potential highlight the fact that the analyzed suspensions remain stable after 28 days. In addition, as far as we know, our study is the first to prove the downregulation of MCM-2 expression in Caco-2 cells exposed to dextran-coated IONPs conjugated with 5-FU. The nanoformulation with the ratio IONP:5-FU 1.5:1 had the highest anti-tumor efficiency, showing a higher accumulation of nanoparticles within the cells, amplified production of ROS, and more potent inhibition of proliferation. Our findings indicate that iron ions might play a role in the enhancement of 5-FU antitumoral activity, thereby setting the premise for a novel approach to improve the 5-FU-based treatment of colorectal cancer.

## Figures and Tables

**Table 1:** The hydrodynamic diameters at different storage times.

Time (Days)		1	7	14	28
D_h_	IONP:5-FU 0.5:1	38.6	42.4	45.8	46.7
	IONP:5-FU 1:1	44.8	48.6	55.5	56.8
	IONP:5-FU 1.5:1	57.6	63.9	66.2	69.4

**Table 2:** The values of zeta potential.

Time (Days)		1	7	14	28
ζ	IONP:5-FU 0.5:1	−39.6	−38.9	−37.9	−36.8
	IONP:5-FU 1:1	−40.5	−39.9	−38.3	−37.7
	IONP:5-FU 1.5:1	−41.9	−41	−40.6	−39.9

## Data Availability

Not applicable.

## References

[1] Siegel R.L., Wagle N.S., Cercek A., Smith R.A., Jemal A. (2023). Colorectal cancer statistics, 2023. CA Cancer J. Clin..

[2] White A., Ironmonger L., Steele R.J.C., Ormiston-Smith N., Crawford C., Seims A. (2018). A review of sex related differences in colorectal cancer incidence, screening uptake, routes to diagnosis, cancer stage and survival in the UK. BMC Cancer.

[3] Jasperson K.W., Tuohy T.M., Neklason D.W., Burt R.W. (2010). Hereditary and Familial Colon cancer. Gastroenterology.

[4] Ishimaru K., Tominaga T., Nonaka T., Fukuda A., Moriyama M., Oyama S., Ishii M., Sawai T., Nagayasu T. (2021). Colorectal cancer in Crohn’s disease-a series of 6 cases. Surg. Case Rep..

[5] Ye P., Xi Y., Huang Z., Xu P. (2020). Linking Obesity with Colorectal Cancer: Epidemiology and Mechanistic Insights. Cancers.

[6] Oruç Z., Kaplan M.A. (2019). Effect of exercise on colorectal cancer prevention and treatment. World J. Gastrointest. Oncol..

[7] Deng Y., Wei B., Zhai Z., Zheng Y., Yao J., Wang S., Xiang D., Hu J., Ye X., Yang S. (2021). Dietary-Related Colorectal Cancer Burden: Estimates From 1990 to 2019. Front. Nutr..

[8] Gran I.T., Park S.-Y., Wilkens L.R., Haiman C.A., Le Marchand L. (2020). Smoking-Related Risks of Colorectal Cancer by Anatomical Subsite and Sex. Am. J. Epidemiol..

[9] Rossi M., Anwar M.J., Usman A., Keshavaezian A., Bishehsar F. (2018). Colorectal Cancer and Alcohol Consumption-Populations to Molecules. Cancers.

[10] Xi Y., Xu P. (2021). Global colorectal cancer burden in 2020 and projections to 2024. Translational. Oncol..

[11] Kotani D., Oki E., Nakamura Y., Yukami H., Mishima S., Bando H., Shirasu H., Yamazaki K., Watanabe J., Kotaka M. (2023). Molecular residual disease and efficacy of adjuvant chemotherapy in patients with colorectal cancer. Nat. Med..

[12] Vodenkova S., Buchler T., Cervena K., Veskrnova V., Vodicka P., Vymetalkova V. (2020). 5-fluorouracil and other fluoropyrimidines in colorectal cancer: Past, present and future. Pharm. Ther..

[13] Okumura K., Shiomi H., Mekata E., Kaizuka M., Endo Y., Kurumi Y., Tani T. (2006). Correlation between chemosensitivity and mRNA expression of 5-fluorouracil-related metabolic enzymes during liver metastasis of colorectal cancer. Oncol. Rep..

[14] Sara J.D., Kaur J., Khodadadi R., Rehman M., Lobo R., Chakrabarti S., Herrmann J., Lerman A., Grothey A. (2018). 5-fluorouracil and cardiotoxicity: A review. Ther. Adv. Med. Oncol..

[15] Anaka M., Abdel-Rahman O. (2022). Managing 5FU Cardiotoxicology in Colorectal Cancer Treatment. Cancer Manag. Res..

[16] Krishmaiah Y., Satyanarayana V., Kumar B.D., Karthikeyan R., Bhaskar P. (2003). In vivo pharmacokinetics in human volunteers: Oral administered guar gum-based colon-targeted 5-fluorouracil tablets. Eur. J. Pharm. Sci..

[17] Entezar-Almahdi E., Mohammadi-Samani S., Tayebi L., Farjadian F. (2020). Recent Advances in Designing 5-Fluorouracil Delivery Systems: A Stepping Stone in the Safe Treatment of Colorectal Cancer. Int. J. Nanomed..

[18] Gu C., Le V., Lang M., Liu J. (2014). Preparation of polysaccharide derivates chitosan-graft-poly(ε caprolactone) amphilic copolymer micelles for 5-fluorouracil drug delivery. Colloids Surf. B.

[19] Zheng Y., Yang W., Wang C., Hu J., Fu S., Dong L., Wu L., Shen X. (2007). Nanoparticles based on the complex of chitosan and polyaspartic acid sodium salt: Preparation, characterization, and the use for 5-fluorouracil delivery. Eur. J. Pharm. Biopharm..

[20] Maghsoudi A., Shojaosadati S.A., Farahni E.V. (2008). 5-fluorouracil-loaded BSA nanoparticles: Formulation, optimization and in vitro release study. AAPS PharmSciTech.

[21] Balas M., Constanda S., Duma-Voiculet A., Prodana M., Hermenean A., Pop S., Demetrescu I., Dinischiotu A. (2016). Fabrication and toxicity characterization of a hybrid material based on oxidized and aminated MWCNT loaded with carboplatin. Toxicol. Vitr..

[22] González-Lavado E., Valdivia L., García-Castaño A., González F., Pesquera C., Valiente R., Fanarraga M.L. (2019). Multi-walled carbon nanotubes complement the anti-tumoral effect of 5-fluorouracil. Oncotarget.

[23] Hiremath C., Kariduraganavar M.Y., Hiremath M.B. (2018). Synergistic delivery of 5-fluorouracil and curcumin using human serum albumin-coated iron oxide nanoparticles by folic acid targeting. Prog. Biomater..

[24] Kiamohammadi L., Asadi L., Shivalilou S., Khoei S., Khoee S., Soleimani M., Minaei S.E. (2021). Physical and Biological Properties of 5-Fluorouracil Polymer-Coated Magnetite Nanographene Oxide as a New Thermosensitizer for Alternative Magnetic hyperthermia and a Magnetic resonance Imaging Contrast Agent: In Vitro and In Vivo study. ACS Omega.

[25] Tavares Luiz M., Santos Rosa Viegas J., Palma Abriata J., Viegas F., Testa Moura de Carvalho Vicentini F., Lopes Badra Bentley M.V., Chorilli M., Maldonado Marchetti J., Tapia-Blácido D.R. (2021). Design of experiments (DoE) to develop and to optimize nanoparticles as drug delivery systems. Eur. J. Pharm. Biopharm..

[26] Lal R., Kumar Marwaha R., Pandita D., Dureja H. (2012). Formulation and Optimization of 5-Fluorouracil Loaded Chitosan Nanoparticles Employing Central Composite Design. Drug Deliv. Lett..

[27] Massart R., Dubois E., Cabuil V., Hasmonay E. (1995). Preparation and properties of monodisperse magnetic fluids. J. Magn. Magn. Mater..

[28] Predoi S.-A., Iconaru S.L., Predoi D. (2023). In Vitro and In Vivo Biological Assays of Dextran Coated Iron Oxide Aqueous Magnetic Fluids. Pharmaceutics.

[29] Prodan A.M., Iconaru S.L., Ciobanu C.S., Chifiriuc M.C., Stoicea M., Predoi D. (2013). Iron Oxide Magnetic Nanoparticles: Characterization and Toxicity Evaluation by In Vitro and In Vivo Assays. J. Nanomat..

[30] Prodan A.M., Iconaru S.L., Chifiriuc C.M., Bleotu C., Ciobanu C.S., Motelica-Heino M., Sizaret S., Predoi D. (2013). Magnetic Properties and Biological Activity Evaluation of Iron Oxide Nanoparticles. J. Nanomat..

[31] Ciobanu C.S., Iconaru S.L., Gyorgy E., Radu M., Costache M., Dinischiotu A., Le Coustumer P., Lafdi K., Predoi D. (2012). Biomedical properties and preparation of iron oxide-dextran nanostructures by MAPLE technique. Chem. Cent. J..

[32] Iconaru S.L., Ciobanu C.S., Le Coustumer P., Predoi D. (2013). Structural Characterization and Magnetic Properties of Iron Oxides Biological Polymers. J. Supercond. Nov. Magn..

[33] Balas M., Dumitrache F., Badea M.A., Fleaca C., Badoi A., Tanasa E., Dinischiotu A. (2018). Coating Dependent In Vitro Biocompatibility of New Fe-Si Nanoparticles. Nanomaterials.

[34] Amini-Fazl M.S., Mohammadi R., Kheiri K. (2019). 5-Fluorouracil loaded chitosan/polyacrylic acid/Fe3O4 magnetic nanocomposite hydrogel as a potential anticancer drug delivery system. Int. J. Biol. Macromol..

[35] Ayyanaar S., Bhaskar R., Esthar S., Vadivel M., Rajesh J., Rajagopal G. (2022). Design and development of 5-fluorouracil loaded biodegradable magnetic microspheres as site-specific drug delivery vehicle for cancer therapy. J. Magn. Magn..

[36] Laurent S., Forge D., Port M., Roch A., Robic C., Vander Elst L., Muller R.N. (2008). Magnetic iron oxide nanoparticles: Synthesis, stabilization, vectorization, physicochemical Characterizations, and Biological Applications. Chem. Rev..

[37] Honary S., Zahir F. (2013). Effect of zeta potential on the properties of nano-drug delivery systems—A review (Part 2). Trop. J. Pharm. Res..

[38] Samimi S., Maghsoudnia N., Eftekhari R.B., Dorkoosh F. (2018). Lipid-Based Nanoparticles for Drug Delivery Systems.

[39] Gumustas M., Sengel-Turk C.T., Gumustas A., Ozkan S.A., Uslu B. (2017). Effect of Polymer-Based Nanoparticles on the Assay of Antimicrobial Drug Delivery Systems.

[40] Ebadi M., Saifullah B., Buskaran K., Hussein M.Z., Fakurazi S. (2019). Synthesis and properties of magnetic nan-otheranostics coated with polyethylene glycol/5-fluorouracil/layered double hydroxid. Int. J. Nanomed..

[41] Cortajarena A.L., Ortega D., Ocampo S.M., Gonzalez-García A., Couleaud P., Miranda R., Belda-Iniesta C., Ayuso-Sacido A. (2014). Engineering Iron Oxide Nanoparticles for Clinical Settings. Nanobiomedicine.

[42] Arbab A.S., Wilson L.B., Ashari P., Jordan E.K., Lewis B.K., Frank J.A. (2005). A model of Lysosomal metabolism of dextran coated supermagnetic iron oxide (SPIO)nanoparticles: Implications for cellular magnetic resonance imaging. NMR Biomed..

[43] Cromer Berman S.M., Wang C.J., Orukari I., Levchenko A., Bulte J.W., Walczak P. (2013). Cell motility of neural stem cells is reduced after SPIO-labeling, which is mitigated after exocytosis. Magn. Reason. Med..

[44] Oh N., Park J.H. (2014). Endocytosis and exocytosis of nanoparticles in mammalian cells. Int. J. Nanomed..

[45] Bruschi M.L., de Toledo L.d.A.S. (2019). Pharmaceutical Applications of Iron-Oxide Magnetic Nanoparticles. Magnetochemistry.

[46] Aranda A., Sequedo L., Tolosa L., Quintas G., Burello E., Castell J.V., Gombau L. (2013). Dichloro-dihydro-fluorescein diacetate (DCFH-DA) assay: A quantitative method for oxida-tive stress assessment of nanoparticle-treated cells. Toxicol. Vitr..

[47] Sies H. (2017). Hydrogen peroxide as a central redox signaling molecule in physiological oxidative stress: Oxidative eustress. Redox Biol..

[48] Sun Y., Cheng Z., Liu S. (2022). MCM2 in human cancer: Functions, mechanisms, and clinical significance. Mol. Med..

[49] Mhaidat N.M., Bouklihacene M., Thorne R.F. (2014). 5-Fluorouracil-induced apoptosis in colorectal cancer cells is caspase-9-dependent and mediated by activation of protein kinase C-δ. Oncol. Lett..

[50] Flanagan L., Meyer M., Fay J., Curry S., Bacon O., Duessmann H., John K., Boland K.C., McNamara D.A., Kay E.W. (2016). Low levels of Caspase-3 predict favourable response to 5FU-based chemotherapy in advanced colorectal cancer: Caspase-3 inhibition as a therapeutic approach. Cell Death Dis..

[51] Yang J.W., Zhang Q.H., Liu T. (2018). Autophagy facilitates anticancer effect of 5-fluorouracil in HCT-116 cells. J. Cancer Res. Ther..

[52] Zhou M., Liu X., Li Z., Huang Q., Li F., Li C.Y. (2018). Caspase-3 regulates the migration, invasion and metastasis of colon cancer cells. Int. J. Cancer.

[53] Akay S., Kayan B., Yang Y. (2017). Solubility and Chromatographic Separation of 5-Fluorouracil under Subcritical Water Conditions. J. Chem. Eng. Data.

[54] Poller J.M., Zaloga J., Schreiber E., Unterweger H., Janko C., Radon P., Eberbeck D., Trahms L., Alexiou C., Friedrich R.P. (2017). Selection of potential iron oxide nanoparticles for breast cancer treatment based on in vitro cytotoxicity and cellular uptake. Int. J. Nanomed..

[55] Sahay G., Alakhova D.Y., Kabanov A.V. (2010). Endocytosis of nanomedicines. J. Control. Release.

[56] Miralinaghi P., Kashani P., Moniri E., Miralinaghi M. (2019). Non-linear kinetic, equilibrium, and thermodynamic studies of 5-fluorouracil adsorption onto chitosan–functionalized graphene oxide. Mater. Res. Express.

